# Pulsatile arterial blood pressure mimicking aortic valve opening during continuous-flow LVAD support: a case report

**DOI:** 10.1186/s13019-019-1039-z

**Published:** 2019-12-18

**Authors:** Matthias Paprotny, Frank Ruschitzka, Bernd Lüders, Markus J. Wilhelm, Raed Aser, Dominique Bettex, Andreas J. Flammer, Alain Rudiger, Stephan Winnik

**Affiliations:** 1 University Heart Center Zurich, University Hospital Zurich, Raemistrasse 100, 8091 Zurich, Switzerland; 2 Institute of Anesthesiology, University Hospital Zurich, Zurich, Switzerland

**Keywords:** Mechanical circulatory support, Left ventricular assist device, Invasive arterial blood pressure monitoring, Aortic valve

## Abstract

**Background:**

Left ventricular assist devices (LVAD) have become a common treatment option in advanced heart failure. Lack of aortic valve opening during left ventricular unloading is a common complication and associated with a worse outcome. Maintaining a minimum pulse pressure is an important goal during the early postoperative period after LVAD implantation since it is commonly seen as secure sign of aortic valve opening.

**Aims/objective:**

We report a case of an LVAD-supported patient with early permanent closure of the aortic valve despite a pulse pressure > 15 mmHg at all times following LVAD implantation. We demonstrate how careful assessment of the invasive arterial blood pressure curve can indicate aortic valve closure irrespective of pulsatile blood flow.

**Method:**

A 69-year old male patient with terminal ischemic cardiomyopathy was referred for long-term mechanical circulatory support. Due to mild aortic regurgitation both an aortic bioprosthesis and a continuous-flow left ventricular assist device were implanted. Postoperative echocardiography documented a patent aortic bioprosthesis and an acceptable residual systolic left ventricular contractility. During invasive arterial blood pressure monitoring repetitive transient slight blood pressure decreases followed by slight blood pressure increases coincided with programmed LVAD flushing cycles. Permanent pulsatile flow with a pulse pressure of ≥15 mmHg conveyed systolic opening of the aortic valve. Echocardiography, however, proved early permanent aortic valve closure. In retrospect, transformation of the automated LVAD flushing cycles into visible changes of the arterial blood pressure curve during invasive blood pressure monitoring is indicative of ejection of the complete cardiac output through LVAD itself, and therefore an early clinical sign of aortic valve closure.

**Discussion/conclusion:**

We present this interesting didactic case to highlight caveats during the early postoperative period after LVAD implantation. Moreover, this case demonstrates that careful and differentiated observation of the arterial blood pressure waveform provides crucial information in this unique and growing patient population of continuous-flow LVAD support.

## Background/introduction

Mechanical circulatory support has advanced considerably within the past two decades [1, 2]. As opposed to first-generation pulsatile left ventricular assist devices (LVAD) which mimicked a systole and diastole, similar to native cardiac physiology, current second and third generation continuous (non-pulsatile) flow devices are much smaller and are associated with increased durability [3]. Their impeller pumps generate continuous flow that serves to continuously unload the left ventricle (LV), from where blood flow is directed through an extra-cardiac graft to the ascending aorta. While LV pressure is reduced, there is an increase in pressure in the ascending aorta, creating a continuous transvalvular pressure gradient. Aortic valve (AV) opening occurs only when left ventricular pressure exceeds the pressure in the ascending aorta. Therefore, AV opening is thought to depend on a) residual left ventricular contractility and b) the amount of LVAD support (full vs. partial). Since LVADs are frequently set to full support, specifically when residual left ventricular contractility is poor, the AV often remains closed throughout the cardiac cycle. Over time, this may lead to valve thrombosis, commissural fusion, reduced AV opening area and, in the long run, to permanent AV closure or even aortic regurgitation (AR) [4, 5]. Systolic AV opening, even if intermittent, is associated with reduced thrombogenicity in the aortic root area [6], which in turn may reduce the risk for neurological events, which are among the most devastating complications of long-term LVAD therapy. The impact of AR in patients with continuous-flow LVAD support is controversial [5]. Yet, AR reduces effective cardiac output, thus elevating left ventricular filling pressures, which may lead to recurrent heart failure symptoms [5, 7–10]. Therefore, in the early postoperative phase, measures are taken to maintain systolic AV opening during continuous-flow LVAD support by carefully orchestrating medical inotropic support and the amount of mechanical left ventricular unloading. Routine invasive arterial blood pressure (BP) monitoring allows assessing pulse pressure (systolic – diastolic pressure) on a beat-to-beat basis [11]. A pulse pressure of > 10 mmHg is targeted, as this is supposed to indicate sufficient AV opening [12]. Here we present a case, highlighting the limitations of this monitoring in the early postoperative phase in the intensive care setting.

## Case presentation

**Fig. 1:**
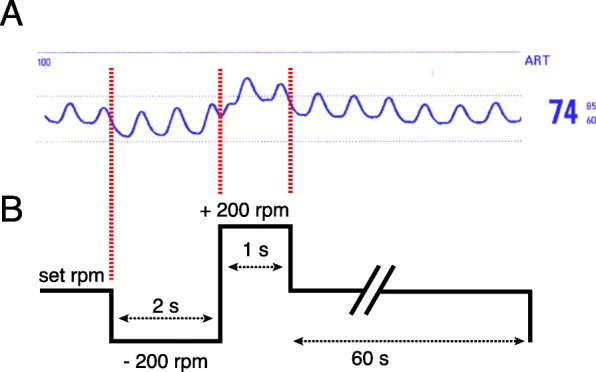
Invasive arterial blood pressure tracing and LVAD flushing cycle. Timely coincidence of a transient arterial blood pressure reduction, followed by a brief increase, as assessed by invasive radial blood pressure monitoring (**a**), with the LVAD flushing cycle (scheme, rpm = rotations per minute) (**b**). Importantly, the flushing cycle transforms into a detectable change in peripheral blood pressure in presence of pulsatile blood flow, indicating left ventricular ejection through the apical inflow cannula and, thus, closure of the aortic valve during the complete cardiac cycle. LVAD = left ventricular assist device

**Fig. 2:**
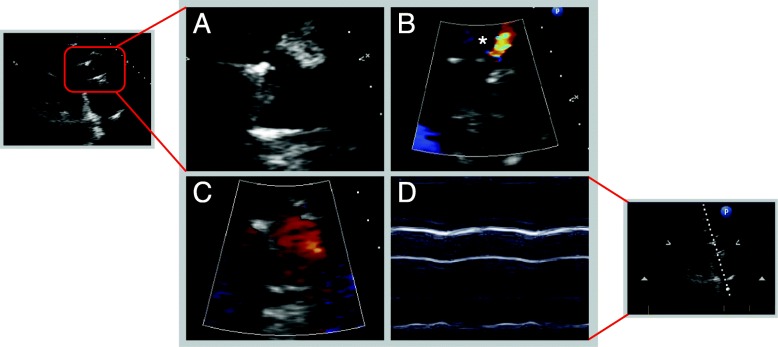
Transthoracic echocardiography. Transthoracic echocardiography on postoperative day 7 showing the closed aortic valve prosthesis in parasternal long axis view (A-D). **a** Zoom on aortic valve prosthesis. **b** Color Doppler showing continuous flow in the extracardiac graft (*) anastomosing into the ascending aorta. **c** Color Doppler showing flow on both sides of the closed aortic valve prosthesis. **d** M-mode tracing without opening of the aortic valve prosthesis throughout the cardiac cycle

A 69-year old diabetic male with ischemic heart disease, severely reduced ejection fraction and surgical revascularization with quadruple coronary artery bypass grafting in 1997 presented to his family physician with nausea and dyspnea for 24 h in late August 2016. A subacute non-ST segment elevation myocardial infarction was diagnosed and partial thrombotic occlusion of the internal mammary bypass graft to the distal left anterior descending coronary artery was identified as culprit lesion. However, the remaining flow was reported to be TIMI grade 3. The venous bypass graft to the first diagonal branch was patent, whereas the remaining venous grafts to the intermediate and the posterior descending artery proved chronically occluded, as did all native coronary vessels. LV enddiastolic pressure (LVEDP) was severely increased (40 mmHg) and LV ejection fraction was below 15%. In spite of increasing congestion of the spontaneously breathing patient the remaining coronary flow was judged sufficient, and a conservative medical management was adjudicated and therapeutic anticoagulation and dual antiplatelet therapy were initiated. After initial recompensation the patient experienced repetitive hemodynamically relevant ventricular tachycardia that could not be controlled with ICD- and medical therapy and was associated with the repetitive need for inotropic support (INTERMACS level 3) [13]. Consequently, the patient was referred to our center for evaluation of advanced heart failure therapy. Due to coexistent severe cerebrovascular disease, chronic renal impairment and his advanced age, the patient was deemed ineligible for transplantation. Decision for permanent mechanical circulatory support (destination therapy) using an LVAD was made. For pre-existent mild aortic regurgitation a bioprosthetic AV (Edwards Perimount Magna 3000) was implanted along with a third-generation, continuous-flow LVAD (HeartWare, Framingham, MA). Initial postoperative echocardiography documented a normally working aortic bioprosthesis. Pulse pressure, as assessed by invasive arterial BP monitoring, was constantly ≥15 mmHg. At postoperative day 7 hemodynamic monitoring showed a repetitive distinct pattern with a brief decrease followed by a transient increase in peripheral arterial BP (Fig. 1a). This pattern occurred every 60 s and coincided with the LVAD flushing cycle. On close observation of the arterial BP waveform, no dicrotic notch was visible between systolic decline and diastolic runoff. Echocardiography testified permanent AV closure despite a residual LV ejection fraction of 15 to 20% (Fig. 2).

## Discussion and conclusion

We present a case of continuous-flow LVAD support in a patient with ischemic cardiomyopathy. Even though systolic contractility of the unloaded LV was sufficient to generate pulsatile blood flow, 7 days after LVAD and bioprosthetic AV implantation, no more systolic AV opening could be documented. This finding was evident by transformation of LVAD flushing cycles into systemic BP changes during preserved pulse pressure ≥ 15 mmHg as assessed by invasive arterial monitoring. The flushing cycle encompasses a reduction of the pump speed by 200 rpm below the set speed for 2 seconds, followed by an increased pump speed to 200 rpm above the set speed for 1 second before the pump returns to the programmed speed (Fig. 1b). This maneuver is routinely applied in order to prevent clot formation and pump thrombosis. Under normal conditions, pulsatile blood flow, generated by residual systolic contraction of the unloaded left ventricle and consecutive ejection through a patent AV, masks these brief LVAD flushing cycles. In the absence of systolic AV opening, systolic ejection is possible through secondary and tertiary, passive LVAD blood flow paths, which are partially independent of impeller position and speed [14]. Thus, LVAD flushing cycles become apparent as visible transient decrease followed by a brief increase in BP on invasive BP monitoring. Transformation of the LVAD flushing cycle into a visible change in peripheral arterial BP in presence of pulsatile blood flow (preserved pulse pressure > 10 mmHg), therefore indicates systolic ejection from the LV exclusively through the apical LVAD while the AV remains closed.

In summary, the presence of pulsatile blood flow alone is insufficient proof of a patent aortic valve during continuous-flow LVAD support. Even in presence of remaining LV contractility, permanent AV closure may occur. However, transmission of LVAD flushing cycles and disappearance of the dicrotic notch in the arterial blood pressure waveform may hint toward AV closure early after continuous-flow LVAD implantation. Careful observation of the arterial blood pressure waveform during the early postoperative phase may provide highly relevant information on both cardiac function and valvular mechanics.

## Data Availability

Not applicable.

## Abbreviations

AR: Aortic regurgitation
AV: Aortic valve
BP: Blood pressure
LV: Left ventricle
LVAD: Left ventricular assist device
LVEDP: Left ventricular enddiastolic pressure
